# mTORC1 Activity in Psoriatic Lesions Is Mediated by Aberrant Regulation through the Tuberous Sclerosis Complex

**DOI:** 10.3390/cells11182847

**Published:** 2022-09-13

**Authors:** Antonio Ferreri, Victoria Lang, Roland Kaufmann, Claudia Buerger

**Affiliations:** Department of Dermatology, University Hospital, Goethe University Frankfurt, 60590 Frankfurt am Main, Germany

**Keywords:** psoriasis, inflammation, mTORC1, tuberous sclerosis complex, cytokines

## Abstract

In the basal, proliferative layer of healthy skin, the mTOR complex 1 (mTORC1) is activated, thus regulating proliferation while preventing differentiation. When cells leave the proliferative, basal compartment, mTORC1 signaling is turned off, which allows differentiation. Under inflammatory conditions, this switch is hijacked by cytokines and prevents proper differentiation. It is currently unknown how mTORC1 is regulated to mediate these effects on keratinocyte differentiation. In other tissues, mTORC1 activity is controlled through various pathways via the tuberous sclerosis complex (TSC). Thus, we investigated whether the TS complex is regulated by proinflammatory cytokines and contributes to the pathogenesis of psoriasis. TNF-α as well as IL-1β induced the phosphorylation of TSC2, especially on S939 via the PI3-K/AKT and MAPK pathway. Surprisingly, increased TSC2 phosphorylation could not be detected in psoriasis patients. Instead, TSC2 was strongly downregulated in lesional psoriatic skin compared to non-lesional skin of the same patients or healthy skin. In vitro inflammatory cytokines induced dissociation of TSC2 from the lysosome, followed by destabilization of the TS complex and degradation. Thus, we assume that in psoriasis, inflammatory cytokines induce strong TSC2 phosphorylation, which in turn leads to its degradation. Consequently, chronic mTORC1 activity impairs ordered keratinocyte differentiation and contributes to the phenotypical changes seen in the psoriatic epidermis.

## 1. Introduction

Psoriasis is a common, chronic inflammatory skin disease that affects 2–3% of the population and is associated with a reduced quality of life and a shortened life expectancy due to the association with the metabolic syndrome and cardiovascular pathologies [1]. Clinically, psoriasis presents with red, scaly plaques, which mostly affect predilection sites such as the extensor surfaces of forearms and shins, umbilical, perianal, retro-auricular regions, and the scalp [2]. These plaques are characterized by epidermal hyper-proliferation with impaired keratinocyte differentiation, extravasation of lymphocytes, and angio(neo)genesis.

In healthy skin, keratinocytes are subject to a strict control between proliferation by asymmetric cell division in the basal layer and ordered terminal differentiation and maturation into corneocytes, forming a tight epidermal barrier. In psoriasis, certain trigger factors (trauma, drugs, infections), together with dysregulated expression of the antimicrobial peptide LL37, are thought to induce sustained activation of plasmacytoid dendritic cells. This process, which normally controls the defense against infection in injured skin, promotes the maturation of myeloid dendritic cells, which induce the differentiation of T cells into Th17 cells through the secretion of interleukin-12 (IL-12) and IL-23. These activated Th17 cells produce effector cytokines such as IL-17A, IL-17F and IL-22, which stimulate keratinocytes to increase proliferation, while differentiation is impaired [3,4]. Activated keratinocytes, in turn, produce important pro-inflammatory cytokines, including TNF-α and IL-1β, which induces a “vicious cycle” of exuberant immune response, epidermal hyper-proliferation and neovascularization, leading to the complex clinical presentation of psoriasis [5].

We previously found that AKT [6], as well as the mTOR kinase and its downstream signaling molecules [7,8] are hyper-activated in psoriatic skin. In healthy skin, mTORC1 signaling is only active in the basal layer and regulates proliferation while preventing differentiation. When cells leave the proliferative compartment, mTORC1 signaling is switched off, which allows for ordered epidermal differentiation. However, pro-inflammatory cytokines activate the PI3K/Akt pathway, which promotes aberrant proliferation [8]. At the same time persistent mTORC1 activation inhibits proper differentiation [8,9]. 

Beyond this model, it is currently unknown how mTORC1 activity is regulated to promote these effects on keratinocyte differentiation. In order to coordinate cell growth and proliferation, the tuberous sclerosis complex (TSC) integrates extracellular cues (e.g., nutrients, energy, oxygen and growth factors) through different kinases such as AKT [10], RSK1 [11] or ERK [12], which regulate mTORC1 activity [13]. This multiprotein complex consists of TSC1/hamartin, TSC2/tuberin and the TBC1 domain family member TBC1D7 [14]. Mutations in TSC2 cause non-malignant tumors in various organs, including the skin, leading to the clinical manifestation of tuberos sclerosis [15]. Under favorable conditions, the mentioned kinases directly phosphorylate TSC at specific serin (S) and threonine (T) residues [16], leading to its dissociation from the lysosome and destabilization of the protein complex [17]. Thus, TSC2, the catalytic subunit of the complex, can no longer act as a GTPase activating protein (GAP) towards the small GTPase RHEB, which remains GTP-loaded and is able to activate mTORC1 on the lysosome [18,19,20]. Activated mTORC1, in turn, regulates key anabolic processes, such as protein biosynthesis through the phosphorylation of S6 kinase-1 (S6K-1) and eukaryotic initiation factor 4E (eIF-4E) binding protein-1 (4E-BP1), and lipid and nucleotide synthesis, and inhibits catabolic processes such as autophagy [9]. In contrast, under unfavorable extracellular conditions i.e., in the absence of growth factors or amino acids, TSC is not phosphorylated, thus remaining at the lysosomal membrane [17,21] and promoting GTP cleavage by RHEB. RHEB is thus unable to activate mTORC1, and anabolic, mTOR-dependent processes fail to occur. 

We hypothesized that the TS complex might also serve as a critical switch to control the mTORC1 function during epidermal maturation. We showed that TSC2 is strongly phosphorylated on S939 by pro-inflammatory cytokines such as TNF-α as well as IL-1β. Blocking the PI3-K/AKT or the MAPK pathway with kinase inhibitors not only reduced TSC Ser939 phosphorylation, but also blocked mTORC1 activity. Surprisingly, we could not find evidence for increased TSC2 phosphorylation in psoriasis patients. Instead, we discovered that TSC2 is significantly downregulated in lesional psoriatic skin when compared to healthy skin or non-lesional skin of the same patients. Additionally, we uncovered, that persistent exposure to inflammatory cytokines such as in psoriasis dislocates the TS complex, inducing its destabilization and degradation, which results in the hyperactivation of mTORC signaling. 

## 2. Materials and Methods

### 2.1. Antibodies and Chemicals

P-TSC2 S939 (LS-C358381) was from Biozol (Sontheim, Germany). Antibodies specific for TSC2/tuberin (#4308), P-AKT S473 (#4060), AKT (#4691), P-S6K T389 (#9234), P-S S235/6 (#2211), S6 (#2217), P-ERK T202/Y204 (#4370), ERK (#4696), P-4E-BP T37/46 (#2855), as well as MG132 and cyclohexamide were from Cell Signaling (Danvers, MA, USA). Involucrin (SY8; ab20202) was from Abcam (Cambridge, UK), Actin antibody (A1978) was from Sigma-Aldrich (St. Louis, MO, USA) and LAMP-2 antibody (sc-18822) from SantaCruz Biotechnology (Dallas, TX, USA). LY294002, U0126 were from were from Calbiochem (San Diego, CA, USA).

### 2.2. Immunohistochemistry

The study was approved by the ethics committee of the Clinic of the Goethe-University (116/11); the Declaration of Helsinki protocols were followed. A total of 10 psoriasis patients between 23–60 years with a confirmed diagnosis of severe plaque-type psoriasis vulgaris for at least 6 months and no current systemic anti-inflammatory therapy gave written informed consent, as did 5 healthy individuals. Punch biopsies (6 mm) from the lesional and non-lesional skin of patients or normal skin of healthy individuals were taken. Specimens were fixed in 4% PFA, and paraffin embedded and 4 μm sections were processed routinely. Primary antibodies were applied overnight, and Histofine Simple Stain AP Multi (Nichirei Bioscience, Tokyo, Japan) was used for detection. Nuclei were stained with hematoxylin. Images were acquired using a Nikon Eclipse Ci microscope (Nikon Europe, Amstelveen, The Netherlands). For semi-quantitative analysis, IHC staining intensity was estimated by two independent investigators on a scale between 0 to 3. Mean values were calculated, and statistical significance was evaluated by one-way ANOVA and uncorrected Fisher’s LSD test.

### 2.3. Cell Culture and Western Blotting

HaCaT keratinocytes [22] were cultured in DMEM with 10% FCS (Life Technologies, Carlsbad, CA, USA). Cells were treated as indicated and lysed in RIPA lysis buffer (Cell Signaling Technology, Dancers, MA, USA). Lysates were adjusted for equal protein amounts, subjected to SDS–PAGE and blotted onto PVDF membranes. After blocking in 5% milk/TBS-T, membranes were probed with the indicated antibodies overnight. Bound antibodies were visualized with HRP-conjugated secondary antibodies using ECL Substrate (Bio-Rad, Hercules, CA, USA).

### 2.4. Immunofluorescence Staining

HaCaT cells were grown on glass coverslips, treated as indicated and formalin fixed. Cells were permeabilized with 0.2% Triton X-100/PBS and blocked with 5% normal goat serum/0.2% Triton/PBS. Primary antibody was applied overnight at 4 °C, and staining with Alexa 488 or Alexa594 labelled secondary antibodies (Life Technologies, Carlsbad, CA, USA) was performed for 1 h at room temperature. Images were acquired through a 100× oil immersion objective on a Nikon Eclipse Ci microscope (Nikon Europe, Amstelveem, The Netherlands). Colocalization of TSC2 and LAMP-2 was quantified using the Coloc2 plugin of Fiji software 2.3.0 (doi:10.1038/nmeth.2019). For each condition, the Manders’ co-localization coefficient using automatic Costes thresholding was calculated from 8 to 14 separate images, each containing between 3 to 10 cells. Mean values were calculated, and the statistical significance was evaluated by one-way ANOVA and uncorrected Fisher’s LSD test.

### 2.5. Statistical Analysis

The mean ± standard error of the mean (SEM) was depicted in the diagrams. Graph Pad Prism 9.4.1 (GraphPad Software, LLC, San Diego, CA, USA) was used for statistical analysis. Multiple groups were compared by ordinary one-way ANOVA followed by an uncorrected Fisher’s LSD test. A *p*-value ≤ 0.05 was considered statistically significant with (* *p* < 0.05, ** *p* < 0.01). 

## 3. Results

### 3.1. Th1 Cytokines Induce TSC2 Phosphorylation via the PI3-K/AKT and MAPK Pathway

**Figure 1:**
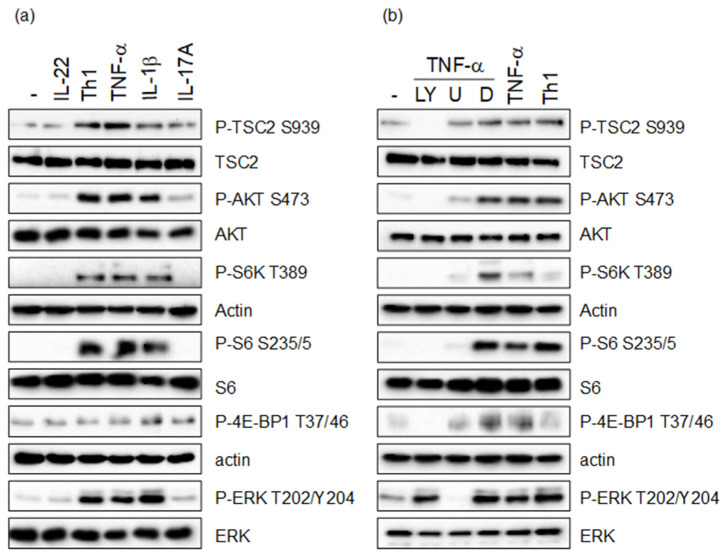
(**a**) HaCaT cells were starved overnight and stimulated with the indicated cytokines (20 ng/mL) or a mix of Th1 cytokines (IL-1β, IL-17A and TNF-α; 20 ng/mL each) for 30 min. (**b**) HaCaT cells were starved overnight and treated for 30 min with the indicated inhibitors (L = 50 μM LY294002; U = 10 μM U0126; D = DMSO), followed by stimulation with 20 ng/mL TNF-α or the Th1 mix (20 ng/mL each). Protein lysates were prepared and analyzed by Western blotting with the indicated antibodies. Equal protein loading was confirmed using total protein antibodies or actin antibody.

To investigate whether cytokine-dependent hyperactivation of mTORC1 is regulated by the tuberous sclerosis complex, we first examined whether TSC2 can be phosphorylated by cytokines involved in the pathogenesis of psoriasis. As we could previously show that AKT is hyper-activated in psoriatic skin, we focused on residues in TSC2, which are mainly regulated by AKT. For four out of the five residues, phosphorylated by AKT (S981, S1130, S1132, T1462), no reliable antibodies could be acquired. Thus, we investigated S939, which is one of the more important residues to control TSC functions towards mTORC1 signaling [10,23,24]. HaCaT keratinocytes were treated with Th1 cytokines with a known effect on keratinocytes during the psoriatic inflammation. As shown previously, IL-17A and IL-22 induced mild phosphorylation of AKT S473, while IL-1β, TNF-α as well as the Th1 mix consisting of IL-1β, IL-17A and TNF-α conferred robust activation of AKT, resulting in strong mTORC1 activation measured by the phosphorylation of the mTORC1 target proteins S6K, S6 and 4E-BP1. At the same time especially TNF-α, as well as the Th1 mix, mediated strong phosphorylation of TSC2 on S939 (Figure 1a). 

To verify that cytokine-dependent phosphorylation of TSC2 is mediated via PI3-K/AKT, HaCaT cells were pre-treated with the PI3-K inhibitor LY294002, which not only inhibited TNF-α-induced AKT activity, but also completely abolished TSC2 phosphorylation. In addition, the MAPK pathway was blocked using the MEK inhibitor U0126, which inhibited TNF-α-induced ERK1 activation, but had only a mild effect on TSC2 phosphorylation (Figure 1b). These results show that pro-inflammatory cytokines of the Th1 spectrum can confer TSC2 phosphorylation via the PI3-K/AKT pathway, and to some degree via the MAPK pathway. 

### 3.2. TSC2 Is Downregulated in Lesional Psoriatic Skin

**Figure 2:**
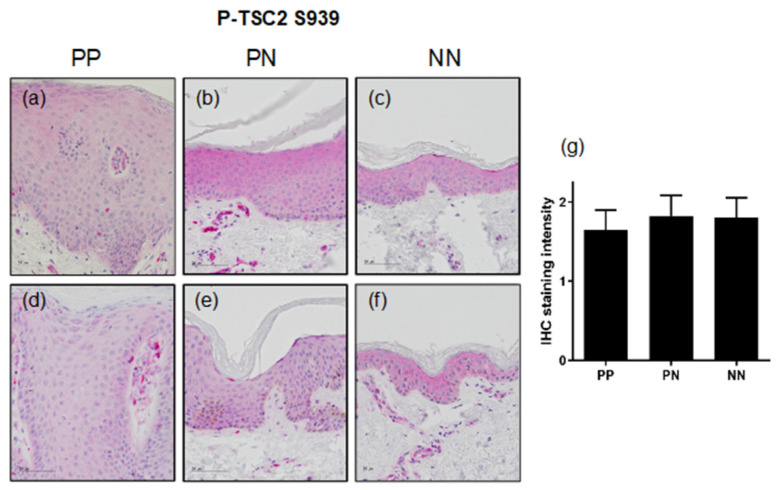
(**a**–**f**) IHC staining with P-TSC2-S939-specific antibodies of two representative biopsies from lesional (PP; **a**,**d**) or non-lesional (PN; **b**,**e**) skin of psoriasis vulgaris patients or healthy donors (NN; **c**,**f**). Bars represent 50 μm. (**g**) The graph represents quantification of IHC staining intensities from 10 psoriasis patients and 3 healthy individuals (mean ± SEM).

To test whether these pro-inflammatory cytokines also mediate the phosphorylation of TSC2 in vivo, non-lesional and lesional psoriatic skin was investigated for TSC2 activation and expression. While healthy skin (NN) displayed the phosphorylation of TSC2 S939 throughout all epidermal layers (Figure 2c,f), non-lesional psoriatic skin (PN) showed comparable levels of TSC2 phosphorylation (Figure 2b,e). Surprisingly, we found that in lesional psoriatic skin (PP), TSC2 did not display enhanced phosphorylation at S939 as expected from the in vitro data, but rather showed similar (Figure 2d) or slightly reduced activation (Figure 2a) compared to non-lesional skin from the same patient. Quantitative analysis of ten patients, underlined this finding, showing slightly but not significantly reduced TSC2 phosphorylation in lesional psoriatic skin (Figure 2g). 

**Figure 3:**
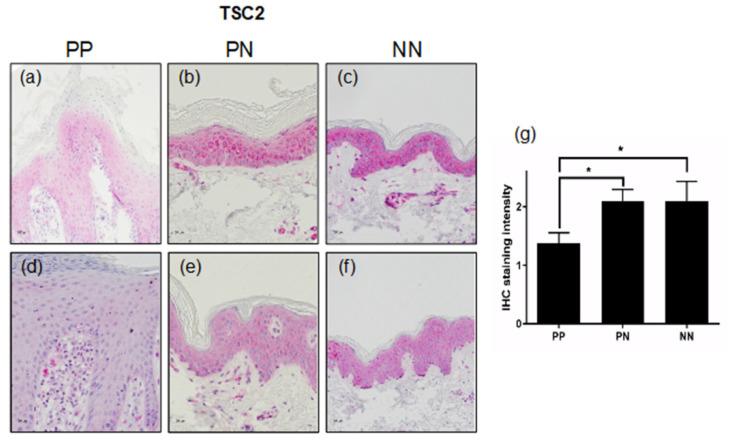
(**a**–**f**) IHC staining with TSC2-specific antibodies of two representative biopsies from lesional (PP; **a**,**d**) or non-lesional (PN; **b**,**e**) skin of psoriasis vulgaris patients or healthy donors (NN; **c**,**f**). Bars represent 50 μm. (**g**) The graph represents the quantification of IHC staining intensities from 10 psoriasis patients and 3 healthy individuals (mean ± SEM). Statistical significance was calculated with one-way ANOVA and Fisher’s LSD test (* *p* < 0.05).

As accumulating phosphorylation of the TS complex on different residues results in the dissociation of TSC from the lysosome and ubiquitin-dependent degradation. We next investigated whether TSC is destabilized in psoriatic skin. We found that healthy skin (NN; Figure 3c,f) as well as non-lesional skin (PN; Figure 3b,e), displayed homogeneous expression of TSC2 throughout the epidermis. However, lesional psoriatic skin (PP; Figure 3a,d) showed a reduced expression of TSC2 in all epidermal layers compared to non-lesional skin from the same patient (Figure 3b,e). This finding was further substantiated by quantifying the staining intensities of all ten psoriasis samples. Psoriatic skin showed a significantly reduced expression of TSC2 in comparison to non-lesional or healthy skin (Figure 3g). Unfortunately, the level of TSC1 being in a complex with TSC2 in the epidermis could not be determined due to the lack of a TSC1-specific, IHC-compatible antibody.

### 3.3. Pro-Inflammatory Cytokines Regulate TSC Function by Spatial Re-Distribution and Degradation

**Figure 4:**
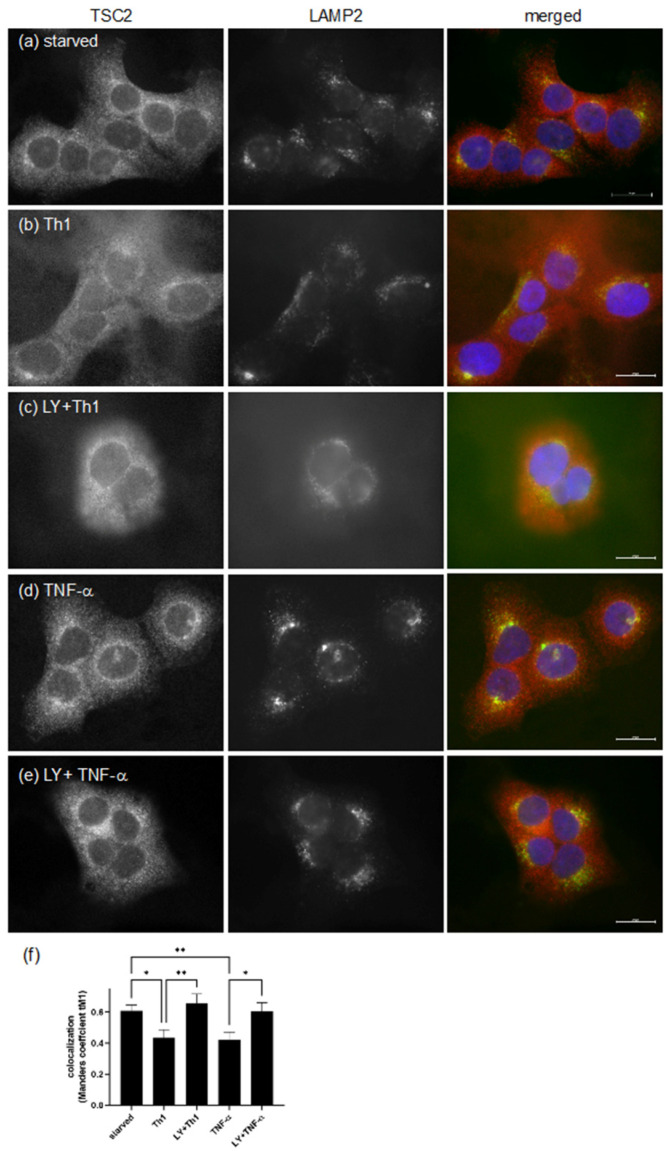
HaCaT cells were serum-starved (**a**) and treated with a cytokine cocktail consisting of IL-1β, IL-17A and TNF-α (20 ng/mL each) (**b**,**c**) or TNF-α alone (20 ng/mL) (**d**,**e**) for 30 min. If indicated, cells were pre-treated with 50 μM LY294002 (LY) for 30 min (**c**,**e**). Cells were fixed and stained for TSC2 (red) and LAMP2 (green), and nuclei were visualized with DAPI (blue). Monochrome images of the red and green channel as well as merged images are shown. Bars indicate 20 μm. (**f**) Colocalization analysis of TSC2 and LAMP2 (thresholded Manders coefficient) is shown as mean ± SEM of 8–14 images from three individual experiments. Statistical significance was assessed with one-way ANOVA and Fisher’s LSD test (* *p* < 0.05, ** *p* < 0.01).

To test whether pro-inflammatory cytokines impair TSC function in psoriatic skin by delocalization and degradation as known from other tissues, HaCaT keratinocytes were stimulated with the Th1 mix or TNF-α, and the localization of TSC2 was visualized by immunofluorescence staining. In starved cells, TSC2 was localized to the lysosomal membrane as indicated by co-staining with the lysosomal marker LAMP2 (Figure 4a). Thus, TSC2 remained in the vicinity of RHEB, supporting its GTPase activity, so that mTORC1 was not activated. However, Th1 cytokines (Figure 4b) or TNF-α (Figure 4d) stimulated dissociation from the lysosomal surface, indicated by diffuse red TSC2 staining, and less yellow co-localization could be detected. This effect was mediated via the cytokine-dependent stimulation of the PI3-K/AKT axis, as the inhibition of PI3-K with LY294002 prevented AKT-mediated TSC2 phosphorylation, and TSC2 remained at the lysosome (Figure 4c,e). Co-localization analysis of several similar images confirmed re-localization of TSC2 into the cytoplasm after an inflammatory stimulus, as the Manders coefficient decreased under these conditions (Figure 4f). Thus, we propose that the psoriatic inflammation drives TSC2 phosphorylation, resulting in its spatial re-distribution, which contributes to TSC2 degradation. 

**Figure 5:**
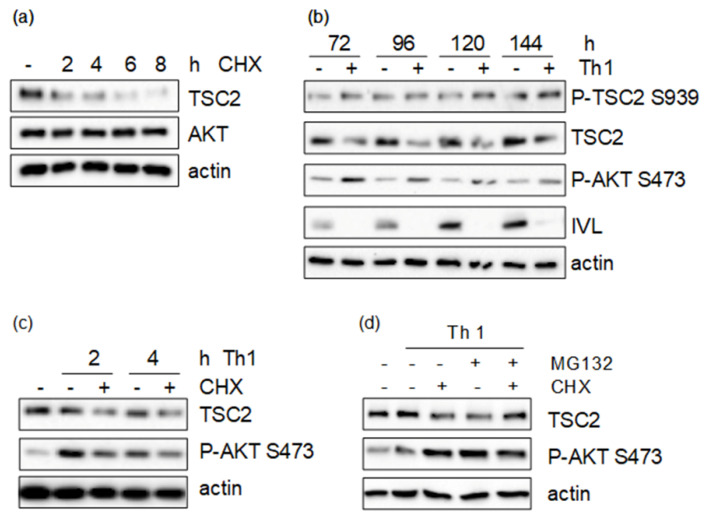
(**a**) HaCaT cells were treated with 10 μg/mL cycloheximide (CHX) for the indicated time points. (**b**) Cells were treated with a Th1 cytokine cocktail (IL-1β, IL-17A, TNF-α 20 ng/mL each) for the indicated time points. (**c**) HaCaT cells were serum-starved overnight and treated with Th1 cytokine and 10 μg/mL cycloheximide (CHX) as indicated for 2 or 4 h. (**d**) Serum-starved cells were treated with Th1 cytokine mix, 10 μg/mL cycloheximide (CHX) or 10 μM MG132 as indicated for 4 h. Protein lysates were prepared and subjected to Western blotting, followed by detection of the indicated proteins.

To investigate this, we first examined the stability of the TSC2 protein by treating HaCaT cells with cycloheximide (CHX), which blocks general protein translation. We could show that TSC2 levels are starting to decline after two to four hours of treatment and are completely absent after eight hours. In contrast, other proteins of the pathway, such as AKT, are unaffected by CHX treatment, thus not being regulated though protein stability (Figure 5a). As we hypothesized that proinflammatory cytokines impair TSC2 stability, cells were treated for these time points with Th1 cytokines, but no impact on TSC2 levels could be seen. However, chronic cytokine treatment for 72 h or longer led to massively reduced TSC2 levels (Figure 5b). Taking the reduced levels of TSC2 in the cytokine-treated samples into account, TSC2 was hyperphosphorylated even after this prolonged treatment. As chronic inflammation interferes with epidermal maturation, the differentiation marker involucrin was assessed to monitor the effect of the cytokines. During the course of the experiment, involucrin accumulated in the untreated samples as keratinocytes grew post-confluently, leading to differentiation, while chronic cytokine exposure blocked the expression of the differentiation marker (Figure 5b). To verify that this cytokine-mediated effect on TSC2 is due to reduced protein stability, we inhibited the 26S proteasome using MG132, which efficiently reduces the degradation of ubiquitin-conjugated proteins. As keratinocytes did not tolerate this inhibitor for the time periods needed for cytokine-induced TSC2 degradation, we established a protocol where keratinocytes were serum-starved and then treated with CHX as well as the Th1 mix. Under these conditions, pro-inflammatory cytokines were able to induce massive degradation of TSC2 already after two and four hours of treatment (Figure 5c). If, under these conditions, the proteasome was inhibited by MG132, a rescue of cytokine-induced TSC2 degradation could be detected (Figure 5d). This provides evidence that inflammation-induced TSC2 phosphorylation likely leads to its ubiquitinylation and degradation via the proteasome.

In summary, we could show that the TS complex is significantly involved in mTORC1 hyperactivation in inflammatory skin diseases, as pro-inflammatory cytokines induce the phosphorylation of TSC2 at S939, which in turn favors its dislocation from the lysosome, followed by degradation. This is underlined by the finding that in psoriatic skin, reduced TSC2 levels could be detected. 

## 4. Discussion

We provide evidence that the TS complex might serve as a crucial switch during epidermal maturation. Under inflammatory conditions such as in psoriasis, it is significantly downregulated, leading to persistent mTORC1 activation, which in turn hampers epidermal maturation.

The TSC1/2 complex has emerged as a central sensor and signal integrator of mTORC1 signaling, being a target for several protein kinases that respond to extracellular cues such as energy, nutrients, growth signals and stress [16]. Especially well described is the regulation through AKT and ERK signaling. We could show that both pathways are activated by cytokines, with a proven role in psoriasis, leading to TSC2 phosphorylation on S939. We and others could show that this residue is a direct target of AKT [24]. In addition, ERK activates RSK1, which has overlapping target sites with AKT such as TSC2 S939 [16]. Thus, chronic exposure to pro-inflammatory cytokines in psoriasis leads to enhanced phosphorylation, which represents a negative signal towards mTORC1. It has been recently found that mTORC1 signaling events are localized at the lysosomal surface [25]. In the absence of any anabolic signals, such as growth factors or amino acids, the TS complex translocates to the lysosome, where TSC2 serves as a GAP for the small GTPase RHEB, thus blocking mTORC1 activation [17,21]. We could show that psoriatic cytokines are able to direct TSC2 away from the lysosomal surface, thus allowing constant mTORC1 activation. Lysosomes are especially important for the formation of a protective epidermal barrier and the formation of corneocytes. Corneocytes are dead cells that mainly serve as a shell for highly insoluble cornified envelop proteins. Thus, keratinocytes eliminate intracellular material and organelles via the lysosomal pathway [26,27]. Interestingly, in HaCaT cells treated with a similar mix of psoriatic cytokines, as well as in psoriatic skin, an altered number of lysosomes could be detected [28], and lysosomal function was impaired [29]. This could imply that under inflammatory conditions detrimental changes in the lysosomal signal platform also affect mTORC1 signaling.

As an additional regulatory mechanism, the phosphorylated TS complex is not only recruited away from the lysosomal mTORC1 signaling complex but is also regulated by protein stability. TSC2 can be bound by the ubiquitin-ligases HERC1 [30], PAM [31], E6AP [32] or TRIM6 [33], becoming highly ubiquitinated and targeted for proteasomal degradation [34]. Furthermore, TSC stability is regulated by the phosphorylation-dependent binding of 14-3-3 [35]. Shumway et al., found that the binding of 14-3-3β inhibits TSC function [36], while it was also described that 14-3-3β binding to the TS complex member TBC1D7 prevents binding of the E3-ubiquitin-ligase β-TrCP1 and thus stabilizes the protein. However, this interaction is only relevant when TBC1D7 is not bound by TSC1, as this interaction stabilizes the whole complex [14,37]. Interestingly, the expression of 14-3-3β and 14-3-3ζ was found to be lower in psoriatic lesions than in healthy skin [38], which would result in increased TSC activity and decreased mTORC1 signaling, which contradicts our previous findings [7,8]. A different report describes 14-3-3β upregulation in psoriatic skin only on the RNA level, but not on the protein level [39]. Thus, a thorough expression analysis of 14-3-3 isoforms in psoriasis and their impact on TSC functions is needed to fully elucidate these mechanisms.

In addition to the presented modes of TSC regulation through phosphorylation, recent work suggests that TSC is also regulated by other post-translational modifications. Gen et al. show that TSC2 is methylated at R1457 and R1459 by the protein arginine methyltransferase 1 (PRMT1), which affects TSC2 stability and interferes with phosphorylation at Thr1462 [40]. In addition, Garcia-Aguilar et al., demonstrate that TSC2 is also acetylated on lysine residues, which promotes its ubiquitination, thus stimulating mTORC1 activity [41]. These secondary modifications are also worth investigating in psoriatic samples. Besides post-translational regulation of the TS complex, the psoriatic inflammation also seems to influence TSC transcription, as gene expression analysis showed the downregulation of TSC1 and 2 in lesional psoriatic skin compared to non-lesional skin [42,43]. 

Thus, an inflammatory milieu as existing in the psoriatic epidermis affects TSC function through different mechanisms, which in turn leads to aberrant mTORC1 activation. mTORC1 hyperactivation not only contributes to enhanced keratinocyte proliferation, leading to epidermal thickening [8], but also interferes with ordered differentiation through the altered expression of keratin 6 [44] and filaggrin [45] as well as reduced nucleophagy [27]. Independent from its role in regulating mTORC1 signaling, the TS complex also contributes to epidermal barrier function by regulating tight junction (TJ) formation. Ablation of TSC1 in mice disrupts TJs and causes a psoriasis-like phenotype. At the same time, junctional TSC1 was markedly reduced in psoriatic skin, suggesting that TSC1 deficiencies underlies epidermal barrier deficiencies due to TJ impairment [46]. 

Hence, the TS complex seems to have divergent levels of regulation that contribute to its localization, stability and activity towards mTORC1 signaling and beyond.

**Figure 6:**
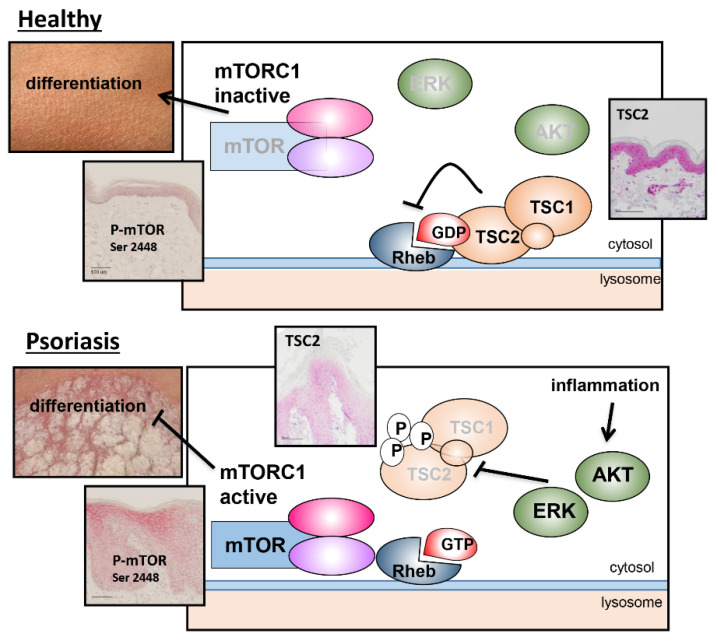
Mechanistic scheme on the regulation of the TS complex in healthy and psoriatic skin.

In summary, we provide the first evidence that the TS complex serves as an important regulator in epidermal differentiation and maturation. Under healthy conditions, in the absence of a signal that stimulates AKT or ERK, TSC2 resides on the lysosomal surface and serves as a GAP for RHEB. This prevents activation of mTORC1 and allows proper keratinocyte differentiation (Figure 6 top panel). Under inflammatory conditions, such as in psoriasis, AKT and ERK become highly activated, leading to the phosphorylation of TSC2, which relocates from the lysosome and is targeted for degradation. This allows for the chronic activation of mTORC1 signaling, which prevents ordered epidermal differentiation and maturation, leading to the phenotypic changes seen in psoriatic skin (Figure 6 lower panel). Consequently, TSC represents as a major switch during epidermal homeostasis, so that its deregulation contributes significantly to the development of skin diseases. Thus, our study supports the notion to further explore the AKT/TSC/mTORC1 pathway as a therapeutic target for the treatment of inflammatory skin diseases. In the past, the mTORC1 inhibitor rapamycin/sirolimus has been used sporadically for systemic therapy due to its immunosuppressive properties, but has been associated with significant side effects [47,48,49]. A single, small clinical trial [50] as well as a mouse study [51], used rapamycin as a topical treatment, resulting in a significant improvement in the clinical score, which support the assumption that the anti-proliferative and differentiation-promoting effects of rapamycin are more important for the therapeutic outcome than its immune-modulatory properties. 

## Data Availability

Not applicable.
